# Frailty assessment using a novel approach based on combined motor and cardiac functions: a pilot study

**DOI:** 10.1186/s12877-022-02849-3

**Published:** 2022-03-14

**Authors:** Nima Toosizadeh, Maryam Eskandari, Hossein Ehsani, Saman Parvaneh, Mehran Asghari, Nancy Sweitzer

**Affiliations:** 1 Department of Biomedical Engineering, University of Arizona, 1230 N Cherry Avenue, Tucson, AZ 85719 USA; 2 Division of Geriatrics, General Internal Medicine and Palliative Medicine, Department of Medicine, University of Arizona, Tucson, USA; 3 Arizona Center on Aging, Department of Medicine, University of Arizona, Tucson, USA; 4 Department of Computer Sciences, University of Arizona, Tucson, AZ USA; 5 Kinesiology Department, University of Maryland, College Park, MD USA; 6 Edwards Life Sciences, Irvine, CA USA; 7 Arizona Sarver Heart Center, Department of Medicine, University of Arizona, Tucson, USA

**Keywords:** Frailty, Heart rate variability, Wearable sensor, Kinematics, Older adults

## Abstract

**Background:**

Previous research showed association between frailty and an impaired autonomic nervous system; however, the direct effect of frailty on heart rate (HR) behavior during physical activity is unclear. The purpose of the current study was to determine the association between HR increase and decrease with frailty during a localized upper-extremity function (UEF) task to establish a multimodal frailty test.

**Methods:**

Older adults aged 65 or older were recruited and performed the UEF task of rapid elbow flexion for 20 s with the right arm. Wearable gyroscopes were used to measure forearm and upper-arm motion, and electrocardiography were recorded using leads on the left chest. Using this setup, HR dynamics were measured, including time to peak HR, recovery time, percentage increase in HR during UEF, and percentage decrease in HR during recovery after UEF.

**Results:**

Fifty-six eligible participants were recruited, including 12 non-frail (age = 76.92 ± 7.32 years), and 40 pre-frail (age = 80.53 ± 8.12 years), and four frail individuals (age = 88.25 ± 4.43 years). Analysis of variance models showed that the percentage increase in HR during UEF and percentage decrease in HR during recovery were both 47% smaller in pre-frail/frail older adults compared to non-frails (*p* < 0.01, effect size = 0.70 and 0.62 for increase and decrease percentages). Using logistic models with both UEF kinematics and HR parameters as independent variables, frailty was predicted with a sensitivity of 0.82 and specificity of 0.83.

**Conclusion:**

Current findings showed evidence of strong association between HR dynamics and frailty. It is suggested that combining kinematics and HR data in a multimodal model may provide a promising objective tool for frailty assessment.

## Introduction

The concept of frailty is used to identify older adults with low physiological reserves, leading to vulnerability to illness, and increased risk of institutionalization and mortality [1, 2]. Muscle loss and weakness (sarcopenia and dynapenia) are the main symptoms of frailty, caused by inflammatory (elevated interleukin 6 (IL-6), C-reactive protein (CRP), tumor necrosis factor alpha (TNFα)), metabolic (deficiencies of various mitochondrial subunits), and hormonal derangements (cortisol and testosterone) that shift homeostasis from an anabolic to a catabolic state [3–11]. Previous research also showed association between frailty and an impaired autonomic nervous system (ANS) because of alterations in electrical conduction and action potential morphology [12, 13]. The presence of a compromised neurohormonal homeostasis associated with frailty as measured by ANS dysfunction is, in turn, associated with health complications and mortality [14–17].

Heart rate variability (HRV: variability in RR intervals) and HR complexity (entropy analysis) during resting have been used for assessing ANS dysfunction and proposed as a vital sign [18–20]. Although resting HRV provides information about abnormal ANS performance, it may not be directly associated with HR increase or recovery in response to physical activities, which show a natural decline with age [21]. Further, between-subject variability exists in resting HR/HRV data (e.g., due to breathing regulation and environmental factors [22–24]). In our previous research we measured HR dynamics defined as HR increase and recovery parameters during and after walking, and investigated the association between these parameters with frailty [25]. We observed that non-frail participants had significantly larger and faster increases in HR during walking, compared to pre-frail/frail older adults [25], more likely due to a lack of cardiovascular reserve and a compromised ANS in pre-frail/frail older adults [26–32]. Although these findings are promising, there are some limitations in assessing HR during walking, including motion artifacts due to whole body movement, lack of space in the clinical settings for performing gait test, and inability of some older adults to walk. Therefore, an alternative physical function testing for assessing HR dynamics was proposed in the current study to address these limitations.

Based on the previous evidence, the aim of the current study, was to establish and validate a platform for simultaneous assessment of motor and cardiac function to assess HR dynamics and predict frailty in community dwelling older adults. For the motor function we have previously validated an upper-extremity function (UEF) test, including rapid elbow flexion, to accurately detect systematic decrements in function, including slowness, weakness, inflexibility, and fatigue [33, 34]. We have validated the UEF motor test for discriminating between frailty groups, among both community dwelling older adults and bed-bound trauma patients, using the Fried frailty index and the short-version Rockwood questionnaire as comparators [35–37]. The hypotheses for the current work were: 1) HR dynamics due to UEF would be significantly associated with frailty; and 2) a combined model including both motor and HR parameters would more strongly be associated to frailty compared to models incorporating only one of these individual measures.

## Methods

### Participants and clinical measures

Older adults were recruited from the primary, secondary, and tertiary health care settings, community providers, assisted living facilities, retirement homes, and aging service organizations between October 2016 and March 2018. Inclusion criteria were: 1) being 65 years or older; and 2) the ability to walk a minimum distance of 9.14 m (30 ft) with or without an assistive device (for the frailty assessment). Exclusion criteria were: 1) severe motor disorders (Parkinson’s disease, multiple sclerosis, or recent stroke); 2) severe upper-extremity disorders (e.g., elbow bilateral fractures or rheumatoid arthritis); 3) cognitive impairment identified by a Mini-Mental State Examination (MMSE) score ≤ 23 [38]; 4) terminal illness (i.e., progressive disease where death within six months is expected as a consequence); 5) diseases/disorders that can directly influence HR (including arrhythmia and use of pacemaker); and 6) usage of β-blockers or similar medications that can influence HR. Written informed consent was obtained according to the principles expressed in the Declaration of Helsinki [39]. The study was approved by the University of Arizona Institutional Review Board.

Clinical measures collected included: 1) MMSE and Montreal Cognitive Assessment (MoCA) for cognition [38, 40]; 2) comorbidity based on Charlson Comorbidity Score (CCI) [41]; and 3) depression using Patient Health Questionnaire (PHQ-9) [42]. These measures were collected because they could potentially influence physical activity and the cardiovascular system performance, and accordingly were considered as adjusting variables in the statistical analysis.

### Frailty assessment

Frailty was assessed using the five-component Fried phenotype as an extensively validated and reliable tool [2]. This frailty test included: 1) self-reported unintentional weight loss of 4.54 kg (10 pounds) or more in the previous year; 2) weakness based on grip strength measurements from both left and right arms (adjusted with body mass index (BMI) and sex) and; 3) slowness based on the required time to walk 4.57 m or 15 ft (adjusted with height and sex); 4) self-reported exhaustion based on a short two-question version of Center for Epidemiological Studies Depression (CES-D) scale; and 5) self-reported low energy expenditure based on a short version of Minnesota Leisure Time Activity questionnaire [43]. Participants were categorized as non-frail if they met none of the criteria, pre-frail if they met one or two criteria, and frail if they met three or more criteria.

### UEF test

Details of UEF validation and index development have been explained comprehensively within our previous work [35–37], and only crucial aspects of UEF regarding the measurement procedure and frailty category assessment were presented here. For UEF, while sitting on a chair, participants performed one trial of full elbow flexion and extension as fast as possible for 20 s using the right arm. Of note, we have shown that UEF results are similar on both sides [35]. Before the test, participants performed a short practice trial with their non-dominant arm to become familiar with the protocol. The protocol was explained to participants, and they were encouraged only once, before elbow flexion, to do the task as fast as possible. To assure consistency, exact same verbal instruction was used, and participants were not further encouraged during the task. Wearable motion sensors (triaxial gyroscope sensors, BioSensics LLC, Cambridge, MA, sampling frequency = 100 Hz) were used to measure forearm and upper-arm motion, and ultimately the elbow angular velocity.

The elbow angular velocity signals from the sensors were filtered to remove noise and drift (first-order high pass butter-worth filter with a cutoff of 2.5 Hz) [44]. Using a peak detection algorithm, maximums and minimums of the angular velocity signal, and subsequently, elbow flexion cycles were detected. Motor function outcomes were derived for each cycle and the average across the 20-s task was calculated. Function outcomes included slowness (speed of elbow flexion), flexibility (range of motion), weakness (strength of upper-extremity muscles), speed variability (motor accuracy), speed reduction (fatigue), and flexion number. For each of the above parameters a subscore was assigned based on previously determined ranges for the the frailty groups (based on the Fried frailty criteria). These subscores were determined previously based on parameter estimate values within multivariable ordinal logistic models, with the Fried frailty categories as the dependent variable and UEF parameters plus demographic information as independent variables [45]. The normalized UEF motor score (range: resilient = 0; extremely frail = 1) for a given participant was then calculated as the sum of subscores corresponding to performance results and demographic information (i.e., BMI score) [37]. The repeatability of UEF motor score was previously tested among a subsample of 14 hospitalized adults (age = 63 ± 12), while UEF was performed twice within (1.1 ± 1.1) days [46]. These findings suggested an excellent repeatability for the UEF motor score indicated by an intraclass correlation coefficient (ICC) of 0.84.

### HR assessment

HR was measured using a wearable system with synchronized electrocardiogram (ECG) and accelerometer sensors (360° eMotion Faros, Mega Electronics, Kuopio, Finland; ECG sampling frequency = 1000 Hz and accelerometer sampling frequency = 100 Hz). One channel ECG was recorded using two electrodes. Electrodes were placed on the left chest, one on the upper mid-thorax, and the other one inferior to the left rib cage. Using the synchronized accelerometer data, the exact starting and endpoints of the UEF task were selected. Then a period of 5 s before and 10 s after the activity were selected, respectively, as baseline and recovery periods. To extract RR intervals, QRS peak detection was performed using the Pan-Tompkins algorithm [47], and detected peaks were manually inspected by two researchers (NT and ME).

Two types of HR measures were extracted, representing: 1) resting-state HR and HRV during baseline; and 2) HR dynamics including HR increase during UEF and HR recovery after UEF. HR baseline parameters included: 1) HR mean; 2) beat-to-beat (RR) interval mean; 3) RR CV: the coefficient of variation (standard deviation divided by mean) of RR intervals; and 4) RMSSD: root mean square of successive heartbeat interval differences. HR dynamics parameters explain the amount and timing of HR changes in response to UEF, which included: 1) time to peak HR: elapsed time to reach maximum HR during the task with reference to minimum baseline HR; 2) HR recovery time: elapsed time to reach minimum HR during the recovery with reference to maximum HR; 3) HR percent/absolute increase: percentage/absolute increase in HR during the task compared to minimum baseline HR; and 4) HR percent/absolute decrease: percent/absolute decrease in HR during the recovery compared to maximum HR during the task.

### Statistical and power analysis

Analysis of variance (ANOVA) models were used to evaluate the differences in demographic parameters between frailty groups, except sex; chi-square (*χ*^*2*^) test was used to assess differences in sex categories among frailty groups. HR parameters were compared between frailty groups using ANOVA models; age, sex, and BMI were considered as covariates, and Cohen’s effect size (*d*) was estimated. Age, sex, and, BMI were selected as adjusting variables, since they have been previously associated with HR measures and frailty [37, 48–50]. ANOVA analyses for comparing HR parameters across frailty groups were repeated with clinical measures with significant association with frailty as covariates. In the next step of the analysis, HR and motor parameters, separately and combined, were used in multiple logistic regression models as independent variables to identify frailty status. A stepwise parameter selection based on Akaike information criterion (AIC) values was implemented to identify predictive independent variables. For each predicting model, the area under the curve (AUC) with 95% CI was calculated using receiver operator characteristics (ROC) curves. Power calculation was performed to detect differences in HR dynamic parameters between frailty groups for the sample size obtained for the current study using G*Power, ANOVA, Fixed-effect, one-way analysis [51].

## Results

### Participants and clinical measures

Fifty-six eligible participants were recruited, including 12 non-frail (age = 76.92 ± 7.32 years), 40 pre-frail (age = 80.53 ± 8.12 years), and four frail (age = 88.25 ± 4.42 years). None of the demographic information was significantly different between the three frailty groups (*p* > 0.10, Table 1). Among clinical measures, CCI comorbidity and PHQ-9 depression scores were significantly different between frailty groups (*p* < 0.03, Table 1).

**Table 1:** Demographic information and clinical measures of participants. A significant difference is identified by the asterisk

Variables	Non-frail (*n* = 12)	Pre-frail(*n* = 40)	Frail (*n* = 4)	*p*-value (effect size)
Male, n (% of the group)	5 (42%)	8 (20%)	2 (50%)	0.20
Age, year (SD)	76.92 (7.32)	80.53 (8.12)	88.25 (4.43)	0.10 (0.55)
Height, cm (SD)	164.36 (9.13)	164.91 (10.18)	157.48 (9.95)	0.97 (0.01)
Weight, kg (SD)	66.58 (14.69)	77.33 (19.49)	57.78 (10.18)	0.15 (0.52)
Body mass index, kg/m^2^ (SD)	24.67 (5.55)	28.20 (5.77)	23.16 (2.01)	0.10 (0.55)
MMSE score, 0–30 (SD)	29.67 (0.65)	29.13 (1.38)	29.25 (0.96)	0.19 (0.50)
MoCA score, 0–30 (SD)	26.25 (3.08)	25.03 (2.90)	23.05 (1.00)	0.15 (0.46)
CCI score, 0–29 (SD)	1.42 (1.78)	3.79 (2.85)	4.50 (3.70)	< 0.01* (1.02)
PHQ-9 score, 0–27 (SD)	0.42 (0.51)	2.15 (2.86)	4.25 (2.87)	0.03* (0.93)
Fried criteria, n (% of the group)				–
Weight loss	0	0 (0%)	1 (25%)	
Weakness	0	14 (35%)	4 (100%)	
Slowness	0	32 (80%)	2 (50%)	
Exhaustion	0	5 (13%)	2 (50%)	
Low energy	0	5 (13%)	3 (75%)	

*SD* standard deviation, *MMSE* Mini-Mental State Exam, *MoCA* Montreal Cognitive Assessment, *CCI* Charlson Comorbidity Index, *PHQ* Patient Health Questionnaire

### UEF motor and HR parameters

**Fig. 1:**
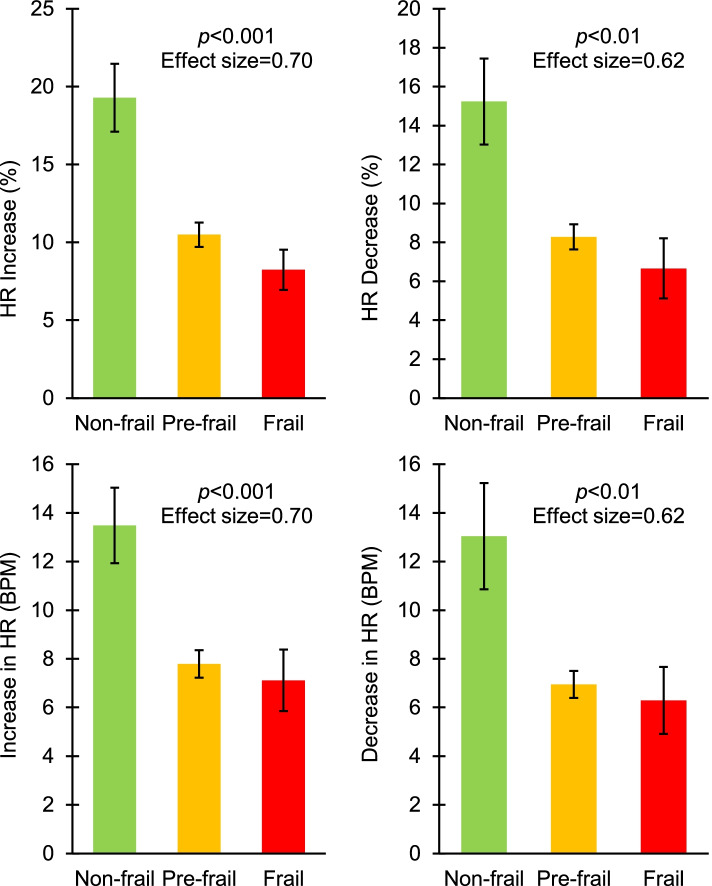
Differences in HR dynamic parameters (percent change and absolute increase/decrease in HR) between non-frail, pre-frail, and frail participants. *p*-values for ANOVA model, adjusted with age, sex, and body mass index are presented

**Fig. 2:**
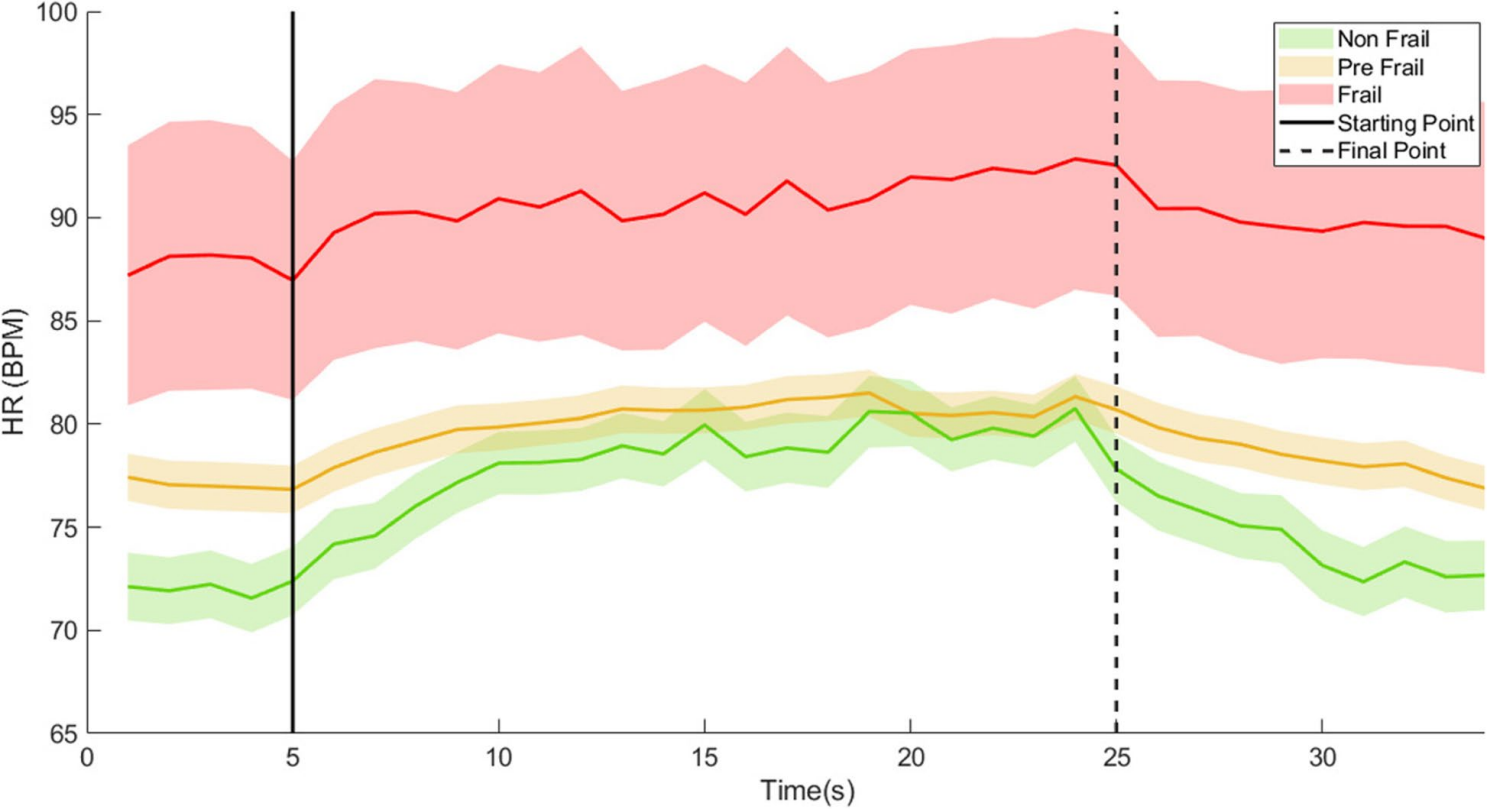
Mean and standard error of HR behavior across frailty groups. Task starting and end point are indicated with vertical lines. For better illustration of changes half of the standard error values are represented. Linear interpolation was used to provide equidistant HR time series across samples

UEF motor score was significantly different between the frailty categories; UEF motor score was 0.32 ± 0.18 on average for non-frail and 0.53 ± 0.23 for pre-frail/frail participants (*p* = 0.04, Table 2). For HR dynamic parameters, pre-frail/frail older adults showed almost half of the amount of HR increase during UEF, and HR decrease during the recovery compared to non-frail participants (*p* < 0.01, Table 2, Figs.1 and 2). The four parameters of HR increase during UEF and HR decrease during recovery were not significantly associated with any of the clinical measures including CCI comorbidity and PHQ-9 depression scores (*p* > 0.11). Further, the association between HR increase and decrease with frailty remained significant when CCI and PHQ-9 were added as covariates (*p* < 0.01). Time to peak and recovery of HR, however, were not significantly different between the frailty groups (*p* > 0.49, Table 2). Although trends of higher HR and smaller HRV for pre-frail/frail were observable, none of the baseline HR parameters were significantly different between the frailty groups (*p* > 0.23, Table 2).

**Table 2:** Results for ANOVA models (adjusted with age, sex, and body mass index), representing differences in UEF motor score and baseline HR and HR dynamics. A significant difference is identified by the asterisk

Parameters	Non-frail (*n* = 12)	Pre-frail (*n* = 40)	Frail (*n* = 4)	*p*-value (effect size)
UEF motor score, 0–1 (SD)	0.32 (0.18)	0.52 (0.24)	0.53 (0.03)	0.04* (0.49)
HR baseline				
HR mean, BPM (SD)	71.52 (11.38)	76.56 (14.69)	97.86 (26.34)	0.23 (0.41)
RR mean, second (SD)	0.86 (0.13)	0.81 (0.14)	0.72 (0.19)	0.26 (0.24)
RR CV, % (SD)	1.70 (1.39)	1.60 (1.56)	1.33 (0.42)	0.93 (0.06)
RMSSD, millisecond	16.80 (18.14)	16.26 (15.39)	11.18 (1.36)	0.90 (0.09)
HR dynamics				
Time to peak HR, second (SD)	16.84 (6.46)	16.00 (5.42)	17.32 (8.25)	0.49 (0.08)
HR recovery time, second (SD)	13.71 (6.22)	14.10 (5.51)	13.42 (9.02)	0.54 (0.04)
HR percent increase, % (SD)	19.28 (7.55)	10.49 (4.93)	8.24 (2.58)	< 0.001* (0.70)
HR percent decrease, % (SD)	15.24 (7.65)	8.28 (4.05)	6.66 (3.09)	< 0.01* (0.62)
Absolute HR increase, BPM (SD)	13.48 (5.38)	7.79 (3.56)	7.12 (2.53)	< 0.01* (0.62)
Absolute HR decrease, BPM (SD)	13.04 (7.57)	6.95 (3.51)	6.29 (2.75)	< 0.01* (0.55)

*UEF* upper-extremity function, *HR* heart rate, *SD* standard deviation, *BPM* beats per minute, *CV* coefficient of variation, *RMSSD* root mean square of successive differences

For logistic model statistical analyses, the pre-frail and frail groups were merged due to the small number of included frail older adults in this study. Results from logistic models showed that percent increase and decrease in HR, as well as UEF motor score were all significantly associated with frailty (*p* < 0.01). Using previously developed UEF motor score in the logistic model, an area under curve (AUC) of receiver operating characteristic (ROC) of 0.78 was achieved. Combining both UEF HR dynamic (i.e., HR percent increase) and motor score, the AUC was improved to 0.87 (Table 3). Using this model, pre-frailty/frailty was predicted with a sensitivity and specificity of 0.82 and 0.83 (Table 3).

**Table 3:** Results for logistic models using HR dynamics and UEF motor score. A significant association is represented by the asterisk

Independent variable	Parameter estimate	Standard error	Chi-square (*χ*^2^)	*p*-value (95% CI)
Model 1 - UEF motor score (AUC = 0.78; AICc = 53.94; Sensitivity = 0.75; Specificity = 0.75)				
Intercept	0.61	0.73	0.70	0.4 (−0.81:2.12)
UEF motor score	−0.05	0.02	6.85	< 0.01 (−0.08:-0.01) *
Model 2 - HR dynamics (AUC = 0.84; AICc = 44.25; Sensitivity = 0.80; Specificity = 0.75)				
Intercept	−4.91	1.27	14.97	< 0.001 (−7.92:-2.81) *
HR percent increase	0.25	0.08	10.38	< 0.001 (0.12:0.44) *
Model 3 - Combined UEF (AUC = 0.87; AICc = 76.67; Sensitivity = 0.82; Specificity = 0.83)				
Intercept	−3.21	1.55	4.28	0.04 (−6.68:-0.45) *
HR percent increase	0.23	0.08	7.73	< 0.01 (0.09:0.42) *
UEF motor score	−0.03	0.02	2.67	0.1 (− 0.07:0.01)

*HR* heart rate; *UEF* upper-extremity function; *AUC* area under curve; *CI* confidence interval; *AICc* Akaike’s information criterion with correction for small sample size

## Discussion

### HR dynamics and frailty

As hypothesized, significant associations were observed between frailty status and HR changes during the activity and afterwards during the recovery period. During physical activity, an increase in sympathetic outflow increases HR and stroke volume to match demand [52, 53]. During recovery from the physical activity, parasympathetic activity increases to reduce HR to baseline [54–56]. Lack of resilience in changing HR in pre-frail/frail older adults can be explained by both a compromised ANS performance or lack of cardiac reserve. Previous research provided evidence of ANS dysfunction with frailty. Focusing on resting state differences in HRV as an indicator of ANS performance, a smaller HRV has been observed among pre-frail and frail older adults compared to non-frails [57]. On the other hand, lack of cardiac reserve during resting, can move pre-frail/frail individuals to a more imbalanced (less homeostatic) and already stressed state, causing an inability to respond to additional stress such as a simple task of arm movement. In confirmation of this theory, although not significant, we observed trends of higher mean HR during resting among pre-frail and frail participants compared to non-frails (Table 3 and Fig. 2).

Only a few studies exist to assess HR dynamics during activity across frailty groups. Smaller changes in HR has been reported previously for lying-to-standing and seated step test [58, 59]. Also, in our previous research we observed that pre-frail/frail older adults had 46% smaller and 49% slower increase in HR during walking compared to pre-frail/frail older adults [46]. One noticeable difference between our previous and current findings is that time to peak HR during activity was significantly different in 15 ft walk test, while this parameter was not different in the current study. One possible explanation is that for pre-frail and frail older adults performing a walking test with a set distance takes longer than non-frails, which consequently may lead to a slower HR increase. This explanation needs to be further assessed by executing walking test with a set duration rather than distance. Nevertheless, based on current findings, assessing changes in HR magnitude, rather than timing of HR changes (both increase and recovery) may provide a more robust way of measuring HR dynamics.

Another important observation was that HR increase can characterize cardiac imbalance behavior, similar or even better than HR recovery. Most previous research has focused on HR recovery for disease diagnosis; studies showed prognostic value in measuring HR recovery one minute after cardiopulmonary exercise testing for heart failure prediction [17, 60]. Nevertheless, all these HR assessments were performed after the physical activity, since performing whole body exercise makes accurate HR assessment complex due to motion artifacts. In the implemented UEF approach, participants performed elbow flexion with the right arm while HR data measurement electrodes were placed on the left side. This ECG placement provided minimal motion artifact from the right-side arm movement to permit accurate dynamic HR assessment.

### Combined HR and motor model

In confirmation of our hypothesis, current results suggest that combining HR and motor function in a single model can enhance frailty prediction in comparison to models involving each of these physiological systems individually. It is believed now that frailty is caused by loss of homeostasis not necessary in one domain, but multiple physiological systems. In other words, frailty is the result of a compromised dynamic interaction between several physiological systems, rather than one specific pathway [61]. Accordingly, the concept of frailty assessment across multiple physiological systems and their interactions has recently drawn more attention. Ghachem et al., for instance, assessed dysregulation of six physiological systems including oxygen transport, kidney/liver function, leukopoiesis, micronutrients, lipids, and electrolytes in association with frailty [62]. They have provided evidence that frailty is more strongly associated with the number of dysregulated systems, rather than the type of dysregulation [62]. In confirmation to previous research, current findings support the hypothesis that assessing multiple physiological systems would improve frailty assessment. Unlike previous work, our approach involved one testing, within which, both cardiac and motor performance were evaluated, to efficiently balance the accuracy and the burden of the testing process.

### Limitation and future direction

Although current findings were promising, there are limitations that warrant future research. First, the sample of community dwelling older adults chosen for the current study was small and may not reflect condition of hospitalized older adults. Due to the small sample of participants, additional analyses were not performed, including assessment of interaction effect of frailty and HR on motor function performance. Further, in the current study the association between baseline HR and HR dynamic parameters were not reported. Since these results were similar to our previous work, we encourage readers to read previously reported findings regarding HR analysis during gait tests [46]. Also, although we validated HR dynamics outcomes for frailty assessment, their test-retest reliability should be investigated in future research.

One other limitation of the current study is the lack of long-term resting HR measurement. Although five seconds of rest before UEF would be enough for short-term HRV assessment, several other analyses related to regularity (complexity analysis) of HR data could not be accomplished here due to limited number of samples for nonlinear dynamic analysis. Previous studies demonstrated significant association between HR complexity and frailty [63–65], and therefore, it would be interesting to explore how the HR complexity during basal condition is related to HR dynamics in response to physical activity, especially across frailty groups. Further, older adult participants with arrhythmia and those who used β-blockers and pacemakers were excluded from the study. Therefore, effects of these disorders and medications on HR measures need to be studies in future. Lastly, none of the participants had identified frail in more than three criteria of the Fried phenotype. Therefore, our results may be limited in presenting too frail older adults. Further, validation using other frailty assessment tools and more importantly within longitudinal studies for predicting adverse outcomes is required in the future.

## Conclusion and clinical implications

Current findings showed that HR changes due to physical activity was smaller among pre-frail and frail individuals during the activity and afterwards during the recovery period compared to non-frail older adults. We also showed that by combining HR and motor function we may improve frailty prediction compared to models incorporating each of these measures individually. The proposed multimodal HR and motor frailty assessment approach is objective and easy to perform. Due to its simplicity, compared to gait test, this test can be performed on hospitalized patients to predict therapy complications and identify patients with treatment-responsive frailty for directing appropriate care, with potential implications for older adults with heart diseases.

## Data Availability

The datasets used and/or analyzed during the current study are available from the corresponding author on reasonable request. The implemented code for HR data analysis will be available for research purposes. Please contact the corresponding author for request access to UEF codes.
